# Novel MEMS Multisensor Chip for Aerodynamic Pressure Measurements †

**DOI:** 10.3390/s25030600

**Published:** 2025-01-21

**Authors:** Žarko Lazić, Milče M. Smiljanić, Dragan Tanasković, Milena Rašljić-Rafajilović, Katarina Cvetanović, Evgenija Milinković, Marko V. Bošković, Stevan Andrić, Ivana Jokić, Predrag Poljak, Miloš Frantlović

**Affiliations:** Institute of Chemistry, Technology and Metallurgy (ICTM), National Institute of the Republic of Serbia, University of Belgrade, Njegoševa 12, 11000 Belgrade, Serbia

**Keywords:** MEMS multisensor, pressure sensing, chip fabrication

## Abstract

The key equipment for performing aerodynamic testing of objects, such as road and railway vehicles, aircraft, and wind turbines, as well as stationary objects such as bridges and buildings, are multichannel pressure measurement instruments (pressure scanners). These instruments are typically based on arrays of separate pressure sensors built in an enclosure that also contains temperature sensors used for temperature compensation. However, there are significant limitations to such a construction, especially when increasing requirements in terms of miniaturization, the number of pressure channels, and high measurement performance must be met at the same time. In this paper, we present the development and realization of an innovative MEMS multisensor chip, which is designed with the intention of overcoming these limitations. The chip has four MEMS piezoresistive pressure-sensing elements and two resistive temperature-sensing elements, which are all monolithically integrated, enabling better sensor matching and thermal coupling while providing a high number of pressure channels per unit area. The main steps of chip development are preliminary chip design, numerical simulations of the chip’s mechanical behavior when exposed to the measured pressure, final chip design, fabrication processes (photolithography, thermal oxidation, diffusion, layer deposition, micromachining, anodic bonding, and wafer dicing), and electrical testing.

## 1. Introduction

Aerodynamic testing of various objects is significant for several scientific and industrial fields, from fluid dynamics to civil engineering and architecture. It is performed on moving and stationary objects, including various types of road and rail vehicles, aircraft and vessels, wind generators, bridges, buildings, and other architectural structures [1,2,3,4,5]. For the purposes of such tests, instruments for multichannel pressure measurement (pressure scanners) are used. In many cases, such equipment is placed inside small objects (e.g., scale models of vehicles) or structural elements (e.g., wind turbine blades), so miniaturization is of key importance. Modern instruments for measuring aerodynamic pressure, intended for aerodynamic testing in wind tunnels or in the open space, are typically based on piezoresistive MEMS pressure sensors. Many advantages of these sensors are important for this application, especially small dimensions, reliability, and low hysteresis. Piezoresistive MEMS pressure sensors are still a large part of the MEMS sensor market today. Although they may be considered a mature technology, new applications continue to appear, and with them, new scientific and technological challenges [6,7,8,9].

Existing pressure scanners typically contain a large number of discrete (single) pressure sensors and one or more discrete temperature sensors in the same housing. Such solutions have the following disadvantages: (1) limited degree of miniaturization in terms of the number of sensing elements (and thus measuring channels) in the available space, (2) limited matching of characteristics between individual sensing elements, and (3) weak thermal coupling between the pressure-sensing elements and temperature-sensing elements, since they have been implemented as separate components.

In this work, our main goal is to overcome the mentioned limitations by developing a multisensor chip that contains several monolithically integrated silicon MEMS piezoresistive sensing elements for pressure measurement and one or more silicon sensing elements for temperature measurement. Our design enables better thermal coupling between pressure- and temperature-sensing elements while providing a higher number of pressure-sensing channels per unit area compared to traditional solutions. Although there are examples of several monolithically integrated sensing elements in the literature, those chips are intended for different purposes and are of a different concept than the chip presented here. For example, contrary to the multisensor chip for multichannel pressure measurements developed in this work, each of the integrated sensors in [10] is intended for the monitoring of a different environmental parameter (temperature, humidity, light intensity, pressure, wind speed, wind direction, magnetic field, or three-axis acceleration). In [11], all the integrated pressure-sensing elements are intended for measuring the same pressure, with the aim of fulfilling the measurement performance requirements of a specific application (radiosonde). For that purpose, there are two groups of sensing elements; each group has a different pressure range, which is used to achieve simultaneous improvements in sensitivity and linearity. Sensing elements within a group have the same characteristics, and their output signals are averaged in order to decrease the measurement error.

After the conceptualization of the multisensor chip, its preliminary design was made. Then numerical simulations were performed in order to investigate the mechanical behavior of the chip’s structure when the measured pressure is applied to one of the pressure sensing elements. The final chip design had been made, and subsequently the first batch of multisensor chips was manufactured and tested.

## 2. Materials and Methods

### 2.1. Main Concepts

Silicon MEMS piezoresistive pressure sensors are based on the piezoresistive effect, i.e., on the change in electrical resistance when the sensor is exposed to the mechanical stress exerted by the applied pressure. The main parts of such sensors are a thin silicon diaphragm and diffused piezoresistors that deform as the diaphragm deflects due to the measured pressure. The multisensor chip described in this paper is developed on the basis of the extensive ICTM’s experience in the field [12,13], particularly the existing commercial SP-12 sensor chip, which was developed for the purposes of measurements in the medium pressure range. It exhibits exceptional measurement linearity not only in its nominal pressure range but also up to the burst pressure. However, the SP-12, as well as other pressure sensors previously developed and manufactured at ICTM for different nominal pressure ranges, has only one pressure-sensing element on the chip.

The multisensor chip contains four sensing elements for pressure measurement and two for temperature measurement, which are integrated together on a silicon substrate. In general, the number of sensing elements on the chip can be higher, which would increase the above-mentioned benefits of the multisensor chip concept even further. However, the chip presented here is the first realization of the concept, so the integration of a larger number of sensing elements on the chip remains to be considered in the future.

Monolithically integrated sensing elements of the multisensor chip are fabricated using the same technological processes, resulting in better matching of the pressure-sensing elements’ characteristics, which can improve the measurement performance in multichannel applications. In our design, the diaphragms of the pressure-sensing elements are square in shape, and each diaphragm has four piezoresistors diffused into it, two in the radial direction and two in the tangential direction relative to the diaphragm’s edges. The piezoresistors are connected in a Wheatstone bridge. The Wheatstone bridge arrangement has the following advantages: (1) since all the resistors in the bridge are piezoresistors, and the resistance of two of them increases with the applied pressure, while the resistance of the remaining two decreases, the combined effect of their resistance change provides higher sensitivity; (2) the differential output voltage of the sensor is close to zero in the absence of the applied pressure, so higher gains can be used in the signal conditioning circuitry, resulting in a higher measurement resolution. When electrical excitation is applied, the differential voltage at the output of the Wheatstone bridge is obtained, and it is proportional to the value of the measured pressure. The two temperature-sensing elements are also monolithically integrated, resulting in the best possible thermal coupling between them and the pressure-sensing elements. As they are used for temperature compensation of the pressure-sensing elements on the chip, this is important for the pressure measurement performance. The basic requirements for the multisensor chip, determined by the target application, are as follows: the nominal pressure range is 10^5^ Pa, the operating temperature ranges from −20 °C to 70 °C, the resistance ranges from 2 kΩ to 3 kΩ at 25 °C, the offset is less than 10% of the full scale, and the excitation current is up to 3 mA (per sensing element).

### 2.2. Numerical Simulations

**Figure 1:**
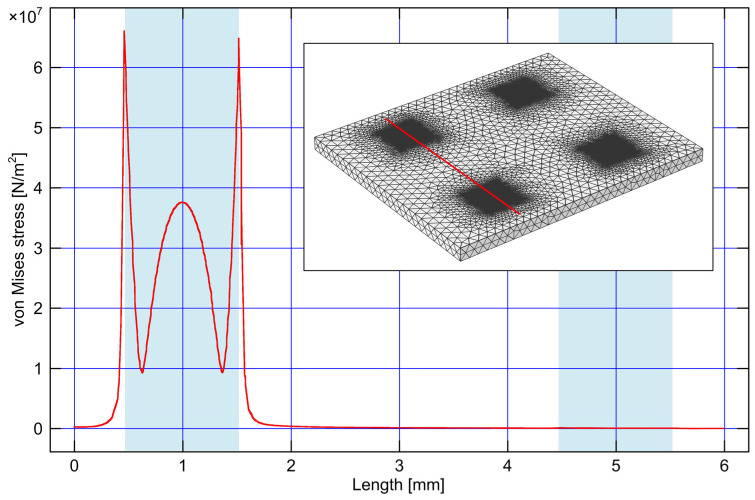
The von Mises stress on the multisensor chip’s top surface caused by a pressure of 100 kPa applied to one sensing element, depending on the position along the line through the middle of the diaphragms of that sensing element and an adjacent sensing element (see the red line in the inset); the light blue areas denote the widths of the two sensing elements’ diaphragms. The inset shows the picture of the chip with the mesh used for simulations.

**Figure 2:**
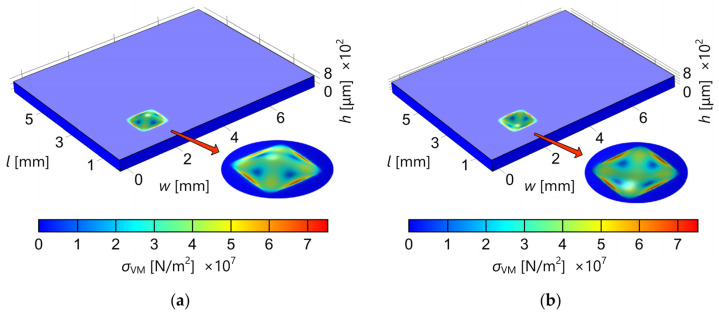
Deflection of the multisensor chip’s top surface caused by a pressure of (**a**) 100 kPa applied to one pressure-sensing element (in the bottom left corner) and (**b**) −100 kPa applied to the same sensing element; color grading represents von Mises stress (the color scale is given on the bottom).

In order to investigate the influence of mechanical stress caused by the pressure applied to one of the pressure-sensing elements on the other sensing elements on the same multisensor chip, we performed a series of numerical simulations based on the finite element method (FEM). The chip is modeled as a rectangular silicon substrate (8.3 mm × 7 mm × 0.4 mm) with four square diaphragms (1 mm × 1 mm × 20 μm) etched from the bottom side of the substrate. The central distance between the adjacent diaphragms is 4 mm. The von Mises stress and the deflection profile of the chip’s top surface were observed in the case of an applied pressure of 100 kPa. The obtained results are presented in graphical form in Figure 1 and Figure 2. Figure 1 shows the dependence of von Mises stress on the position along the line through the middle of the diaphragms of the sensing element with the applied pressure and an adjacent sensing element without the applied pressure (the inset shows the picture of the chip with the mesh used for simulations). Figure 2a shows the deflection of the multisensor chip’s top surface caused by the differential pressure of 100 kPa applied only to the pressure-sensing element in the bottom left corner. Figure 2b also shows the deflection of the chip’s top surface, but in the case of a differential pressure value of −100 kPa. The von Mises stress is represented by color grading according to the given color scale.

The results of the performed simulations confirm that the influence of mechanical stress caused by the pressure applied to one pressure-sensing element on other pressure-sensing elements on the same multisensor chip is negligible. Based on this conclusion, the final design of the multisensor chip was made and subsequently used for fabrication.

### 2.3. Chip Fabrication

Typically, silicon piezoresistive MEMS pressure sensor chips are fabricated on single-crystal silicon wafers with a crystallographic orientation (100). A pressure-sensing element is a micro-electro-mechanical structure that consists of a diaphragm and piezoresistors formed near the diaphragm’s edges in the crystallographic direction <110>. The placement of the piezoresistors is critical for the sensor’s performance. Optimal locations are those with the highest mechanical stress during the diaphragm deflection caused by the measured pressure.

**Figure 3:**
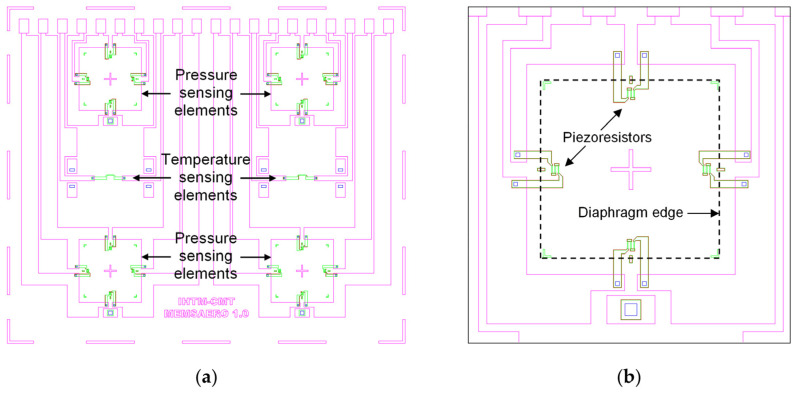
Image of superimposed photolithography masks used for chip fabrication: (**a**) whole chip; (**b**) magnified detail showing one pressure-sensing element.

The layout of the multisensor chip is shown in Figure 3a [14]. Its overall dimensions are 8.3 mm × 7 mm × 0.4 mm. In addition to four pressure-sensing elements and two temperature-sensing elements, it contains metallic interconnects and wire-bonding pads. One of the design requirements was to place all the wire-bonding pads along one edge of the chip in order to facilitate making connections between several chips with electronic circuitry on a printed circuit board. Figure 3b is a magnified detail showing one pressure-sensing element [14]. The dimensions of its diaphragm are 1 mm × 1 mm, and the diaphragm thickness is 20 μm. During the development of the pressure-sensing element structure, optimal locations for the integration of diffused piezoresistors were studied. The location where a piezoresistor is diffused in the square diaphragm of a precisely determined thickness has been optimized by simulations and confirmed experimentally. The piezoresistor’s placement at precisely defined distances from the edge of the diaphragm, in the lateral and tangential directions, results in high sensitivity and exceptional linearity up to the burst pressure.

The chip was produced using 1 μm technology. The photolithography masks were created using the Microtech LaserWriter LW405 (MICROTECH srl, Palermo, Italy). The photolithography mask set consists of five masks:

1. Mask TOP_1 for the diffusion of p-type impurities (boron) for making good Ohmic contacts,
2. Mask TOP_2 for the diffusion of p-type impurities (boron) for the definition of piezoresistors,
3. Mask TOP_3 for the definition of the openings in the protective oxide for metallic contacts,
4. Mask TOP_4 for the definition of metallic interconnections,
5. Mask BOT_1 for the definition of the silicon diaphragm.

The first four masks were processed on the top side of the silicon wafer, while the fifth mask was processed on the bottom side of the wafer.

**Figure 4:**
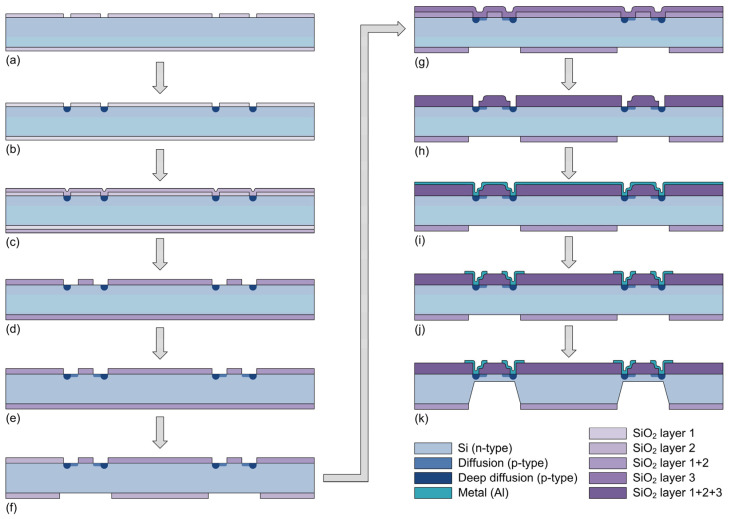
The sequence of the chip fabrication processes: (**a**) etching of SiO_2_; (**b**) deep diffusion of p-type impurities; (**c**) oxidation; (**d**) etching of SiO_2_; (**e**) diffusion of p-type impurities; (**f**) etching of SiO_2_ on the bottom side of the chip; (**g**) oxidation; (**h**) etching of SiO_2_; (**i**) deposition of the Al layer by sputtering; (**j**) etching of Al; (**k**) forming of the pressure-sensing elements’ diaphragms by the etching of Si; (the drawings of the chip are not to scale).

The fabrication of the multisensor chip started with silicon wafers of the following characteristics: n-type (resistivity from 3 Ωcm to 5 Ωcm), double-side polished, 76.2 mm (3′′) in diameter, (100) orientation. The sequence of the chip fabrication processes is shown schematically in Figure 4. The processes will be described in more detail in the following paragraphs.

**Figure 5:**
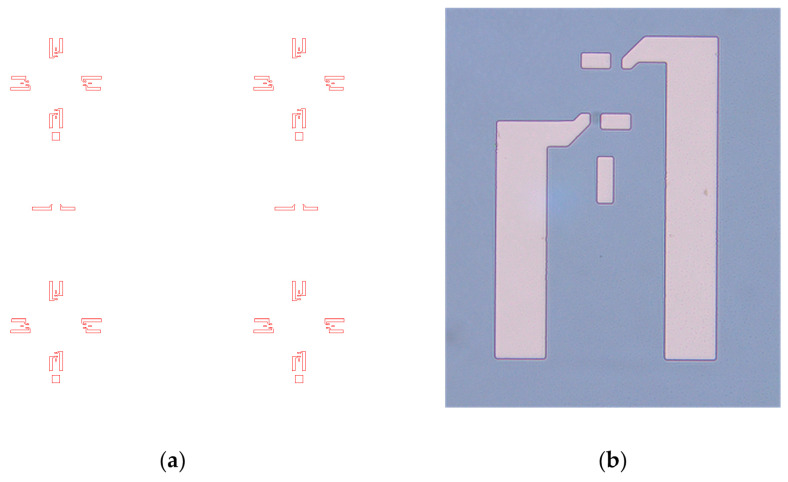
(**a**) Layout of the TOP_1 photolithography mask for the diffusion of p-type impurities for making Ohmic contacts; (**b**) photomicrograph of Ohmic contacts for one piezoresistor on the processed Si wafer after the photolithography step using this mask.

After the thermal oxidation of the silicon wafer, the first photolithographic step with the TOP_1 mask was carried out in order to create openings in the silicon dioxide for the diffusion of p-type impurities (boron). Figure 5a shows the layout of the TOP_1 mask, and Figure 5b shows the photomicrograph of the pattern corresponding to one piezoresistor on the processed Si wafer after the photolithography step using this mask. The silicon dioxide was etched using BHF. 

**Figure 6:**
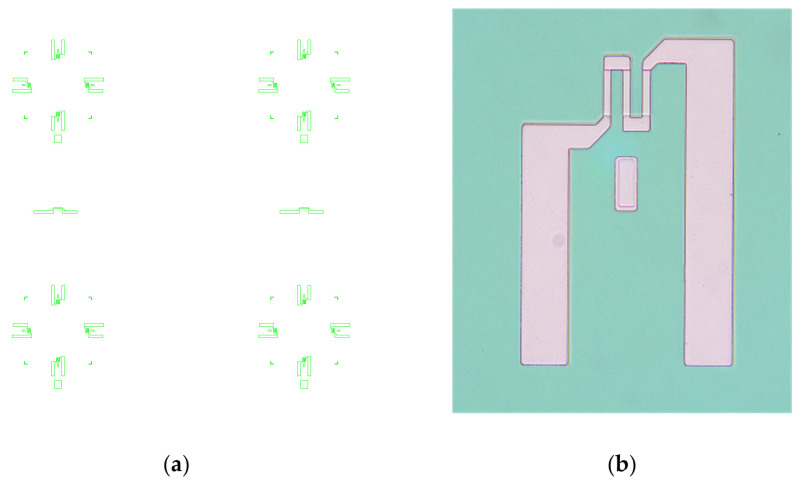
(**a**) Layout of the TOP_2 photolithography mask for the diffusion of p-type impurities for the definition of piezoresistors; (**b**) photomicrograph of one piezoresistor on the processed Si wafer after the photolithography step using this mask.

The next fabrication step was the second thermal oxidation. After that, the second photolithographic step was performed using the TOP_2 mask. This mask defines the piezoresistors. The piezoresistors were formed by the diffusion of p-type impurities (boron), resulting in electrical resistance in the range of 2 kΩ to 4 kΩ. Figure 6a shows the layout of the TOP_2 mask, and Figure 6b shows the photomicrograph of one formed piezoresistor on the processed Si wafer after the photolithography step using this mask. The width of the piezoresistor is 5 μm, and its total length is 135 μm.

**Figure 7:**
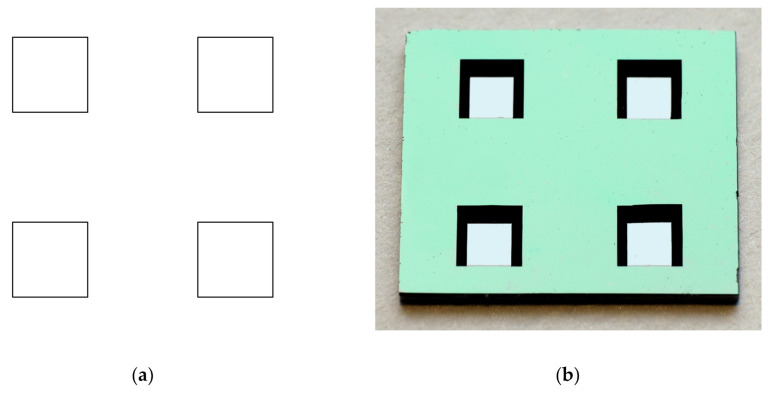
(**a**) Layout of the BOT_1 photolithography mask for the definition of the silicon diaphragm; (**b**) photograph of the bottom side of the chip with etched diaphragms of four pressure-sensing elements.

On the bottom side of the wafer, the photolithographic step using the BOT_1 mask for the definition of the Si diaphragm was performed. This photolithographic step was carried out using the EVG 620 double-sided mask aligner (EV Group Europe & Asia/Pacific GmbH, St. Florian am Inn, Austria). The accuracy of the alignment of masks on the top and bottom sides of the wafer was approximately 1 μm. This step is very important because it defines the position of the piezoresistors near the diaphragm edges. Figure 7a shows the layout of the BOT_1 photolithography mask, and Figure 7b shows the photograph of the bottom side of the fabricated chip, with etched diaphragms of the four pressure-sensing elements.

The piezoresistor diffusion was the last high-temperature step in the fabrication of the multisensor chip. All subsequent processes were performed at low temperatures to prevent changes in the piezoresistors’ resistance. Therefore, the next technological step was the low-temperature sputter deposition of protective silicon dioxide. The deposition was performed using the Perkin-Elmer Sputtering System Model 2400.

**Figure 8:**
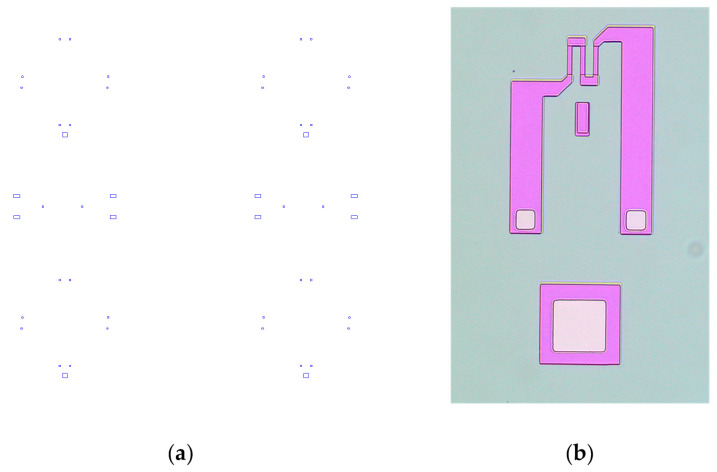
(**a**) Layout of the TOP_3 photolithography mask for the definition of the openings in the protective oxide for metallic contacts; (**b**) photomicrograph of one piezoresistor on the processed Si wafer after the photolithography step using this mask; the light squares are openings in the protective oxide for metallic contacts.

The next photolithography step was then performed using the TOP_3 mask. This mask defines the openings in the protective oxide for metallic contacts. Figure 8a shows the TOP_3 mask’s layout, and Figure 8b is the photomicrograph of one piezoresistor on the processed Si wafer after the photolithography step using this mask.

**Figure 9:**
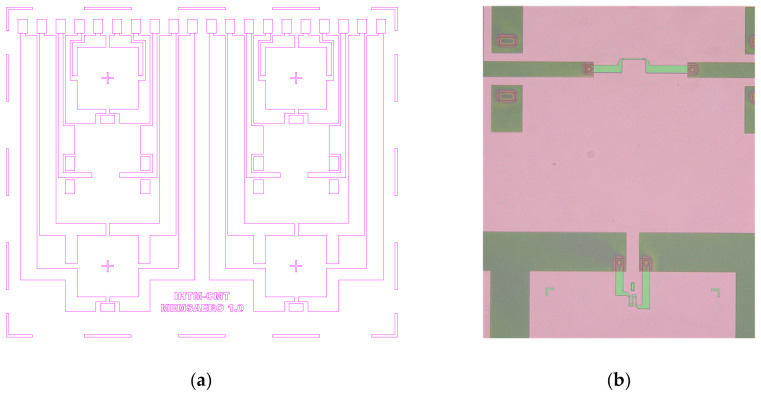
(**a**) Layout of the TOP_4 photolithography mask for the definition of metallic interconnections; (**b**) photomicrograph of a temperature-sensing element (**top**) and a part of a pressure-sensing element (**bottom**) on the processed Si wafer after the photolithography step using this mask; dark green areas are metallic interconnections made by the sputter deposition of aluminum.

Finally, the sputter deposition of aluminum in high-vacuum conditions at the pressure of 2 × 10^−4^ Pa was performed. Then, a photolithography step was performed by using the TOP_4 mask for the definition of metallic interconnections, as shown in Figure 9a,b.

The pressure-sensing elements’ diaphragms were produced using anisotropic wet chemical etching in a 30% KOH water solution at the constant temperature of 80 °C. In order to prevent the aluminum layer deposited on the wafer from being dissolved in the KOH water solution, we used a special tool with back-side protection during the etching process. The tool is a wafer holder (IL-type, AMMT Advanced Micromachining Tools GmbH, Frankenthal, Germany), which has the possibility of temporary illumination from the back side, and the back-side light source is incorporated in the tool. Silicon becomes transparent at thicknesses below about 25 μm, and diaphragms have specific colors for different thicknesses. During the etching process, the thickness of the diaphragms was monitored by observing the diaphragm’s color. The multisensor chip has been designed for the nominal pressure of 10^5^ Pa (1 bar), so the thickness of the diaphragm is 20 μm. 

**Figure 10:**
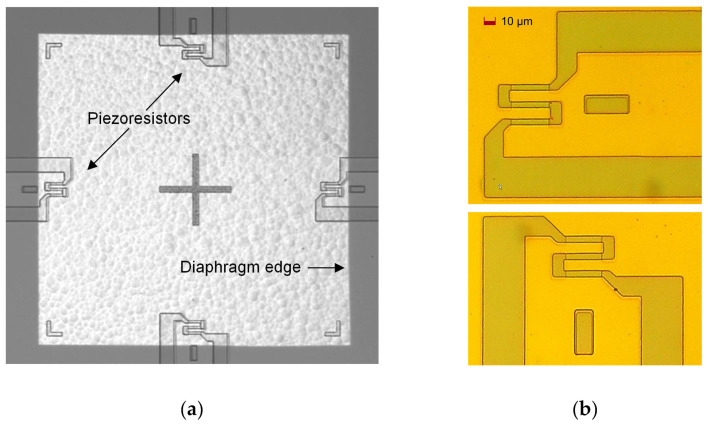
(**a**) Photomicrograph of the etched diaphragm with four piezoresistors (made using an IR camera on the AML AWB-04 Aligner Wafer Bonder); (**b**) photomicrograph of a radial piezoresistor (**top**) and a tangential piezoresistor (**bottom**).

Figure 10a shows the photomicrograph of the etched diaphragm with four piezoresistors, as well as their positions relative to the diaphragm edges. This image was taken in infrared light by using the AML AWB-04 Aligner Wafer Bonder (Applied Microengineering Ltd., Oxfordshire, UK). Figure 10b shows photomicrographs of a radial and a tangential piezoresistor.

In order to obtain separate multisensor chips, the processed silicon wafers were diced using a wafer-dicing machine.

The fabricated multisensor chip is intended for use as a differential pressure sensor in aerodynamic pressure measurements. For that purpose, it must be permanently affixed to a glass substrate. The glass substrate must have the same dimensions as the multisensor chip (except for the thickness of 1 mm) and must have four through-holes at appropriate positions to match the four diaphragms on the multisensor chip. We used borosilicate glass of the Borofloat 33 type (SCHOTT Technical Glass Solutions GmbH, Jena, Germany), with four mechanically drilled through-holes. The multisensor chip was anodically bonded to the glass substrate using the AML AWB-04 Aligner Wafer Bonder.

## 3. Results

**Figure 11:**
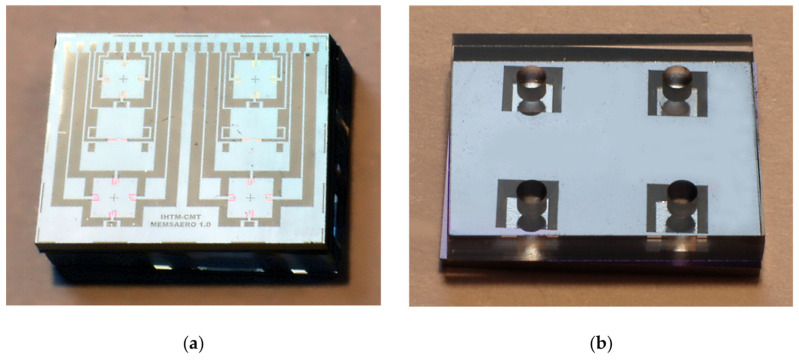
Photographs of the fabricated multisensor chip anodically bonded to a glass substrate: (**a**) top side; (**b**) bottom side.

The photographs of the realized multisensor chip in Figure 11a,b show the top and bottom sides of the fabricated chip anodically bonded to the glass substrate. In total, three silicon wafers were processed, each with 30 chips. Electrical tests of individual chips have been carried out using a probing station and a semiconductor parameter analyzer.

**Figure 12:**
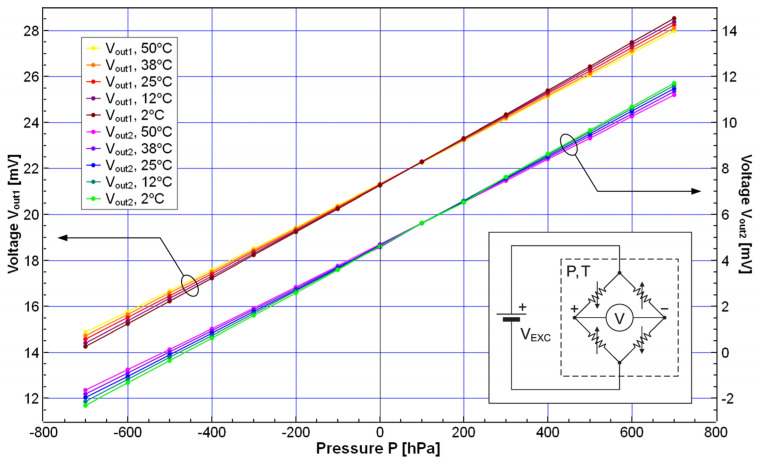
The dependence of the output voltages of two pressure-sensing elements (on the same multisensor chip) on the applied pressure, with temperature as a parameter. Inset: simplified schematic diagram of the measurement setup: the electrical input of a pressure-sensing element (shown as a Wheatstone bridge consisting of four piezoresistors) is connected to the excitation voltage source, and the output to the voltmeter; T and P denote the pressure and temperature to which the multisensor chip is exposed.

Preliminary characterization of the chip in terms of the dependence of the pressure-sensing elements’ output signal on the applied pressure and temperature was performed in the following manner. The pressure-sensing elements were connected to a constant voltage source (Agilent E3649A programmable DC power supply, Agilent Technologies, Inc., Santa Clara, CA, USA) that provided the sensor excitation *V*_EXC_ = 2.5 V. The output voltages of two pressure-sensing elements (*V*_out1_ and *V*_out2_) were measured at several pressure and temperature values (using the Agilent 34410A digital multimeter, Agilent Technologies, Inc., Santa Clara, CA, USA). The schematic diagram is shown in the inset of Figure 12. The applied pressure values were in the range of −700 hPa to 700 hPa (set using the Mensor APC 600 automated pressure calibrator, Mensor LP, San Marcos, TX, USA), and the temperature varied from 2 °C to 50 °C (set using the Heraeus Vötsch VMT 04/140 temperature chamber, Heraeus Vötsch GmbH, Balingen, Germany). The results are presented graphically in Figure 12. It can be concluded from the diagram that, apart from the difference in the output offset values (due to fabrication imperfections), the two sensing elements are well-matched in terms of pressure sensitivity and temperature dependence, which is important for the overall measurement performance of a multichannel system. Our measurement results indicate that the difference in pressure sensitivity is less than 2% (at 25 °C), while in the case of single (discrete) sensors, the difference can be as high as 10%. Additionally, the difference in the temperature dependence of sensitivity is less than 2%, while it can be more than 10 times higher in the case of discrete sensors.

## 4. Conclusions

In this paper, we presented the newly developed MEMS multisensor chip intended for multichannel aerodynamic pressure measurements. First, we explained the main concepts of the multisensor chip, its principle of operation, and specific aspects of its design. We gave an overview of the numerical simulations performed, which were used in order to verify that there are no parasitic mechanical influences between pressure-sensing elements on the chip. After that, the fabrication of the multisensor chip was described in detail, with images that show the most important fabrication steps. Finally, we performed the preliminary characterization of the chip in terms of the dependence of the pressure-sensing elements’ output signal on the applied pressure and temperature. Based on the presented results, it can be concluded that the main objectives of our work have been accomplished.

The development of the multisensor chip presented in this paper is an important step towards higher levels of integration and miniaturization of aerodynamic pressure measurement instruments. In our future work in this field, we will focus on the detailed characterization of the multisensor chip, and on the development of digital signal-processing methods that can further improve the pressure measurement performance. The possibility of increasing the number of sensing elements and their density on the chip will be considered as the next technological challenge.

## Data Availability

The original contributions presented in the study are included in the article, further inquiries can be directed to the corresponding author.
